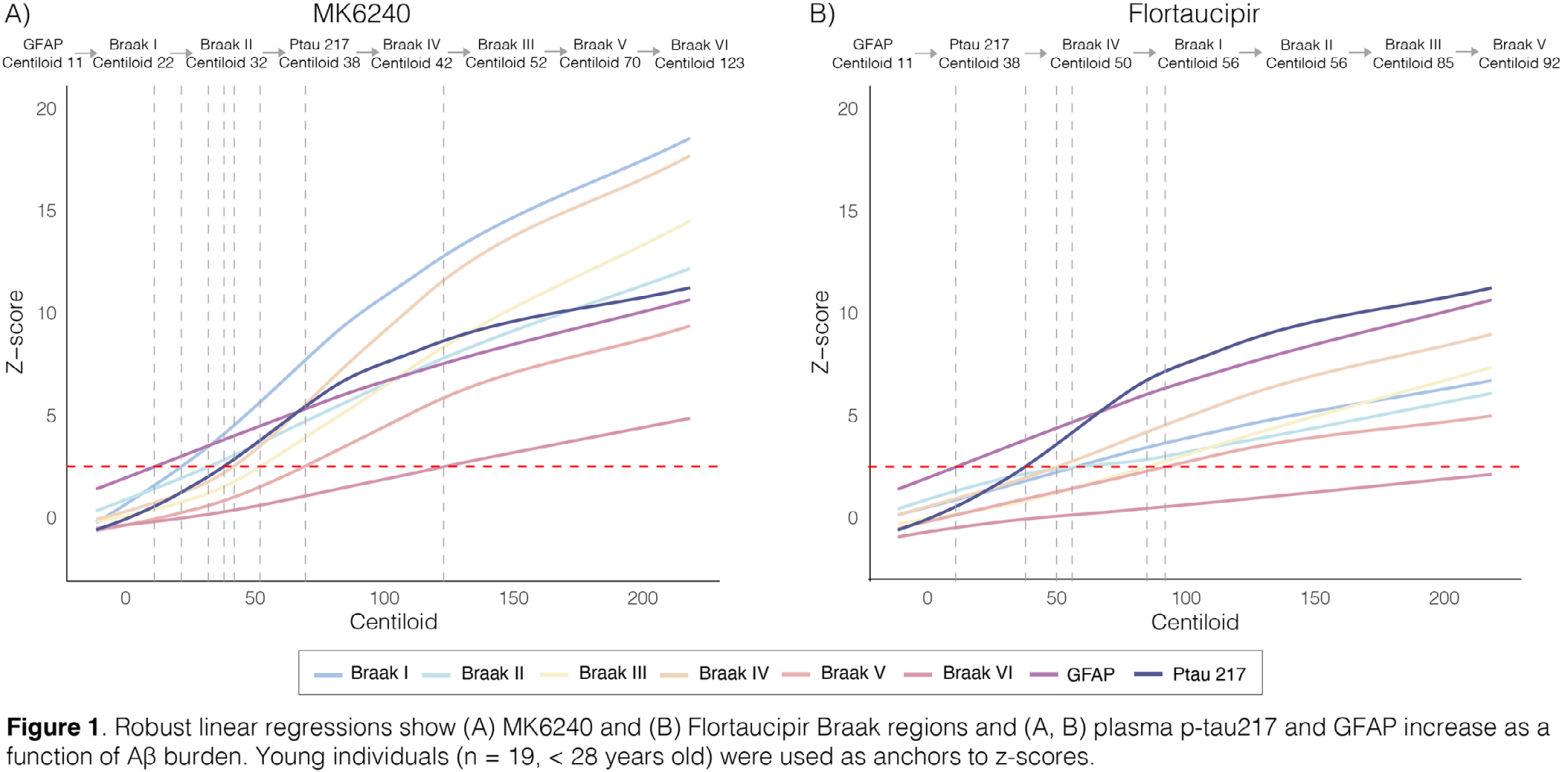# Aβ‐dependent trajectories of Braak stages for MK6240 and Flortaucipir tau PET

**DOI:** 10.1002/alz70856_107040

**Published:** 2026-01-10

**Authors:** Bruna Bellaver, Guilherme Povala, Pamela C.L. Ferreira, Guilherme Bauer‐Negrini, Firoza Z Lussier, Livia Amaral, Andreia Rocha, Joseph C. Masdeu, Dana L Tudorascu, Thomas K Karikari, David N. Soleimani‐Meigooni, Juan Fortea, Val J Lowe, Hwamee Oh, Belen Pascual, Brian A. Gordon, Pedro Rosa‐Neto, Suzanne L. Baker, Tharick A Pascoal

**Affiliations:** ^1^ University of Pittsburgh, Pittsburgh, PA, USA; ^2^ Houston Methodist Research Institute, Houston, TX, USA; ^3^ University of California, San Francisco, San Francisco, CA, USA; ^4^ Sant Pau Memory Unit, Hospital de la Santa Creu i Sant Pau, Biomedical Research Institute Sant Pau, Barcelona, Spain; ^5^ Mayo Clinic, Rochester, MN, USA; ^6^ Brown University, Providence, RI, USA; ^7^ Department of Radiology, Washington University School of Medicine, Saint Louis, MO, USA; ^8^ McGill University, Montreal, QC, Canada; ^9^ Lawrence Berkeley National Laboratory, Berkeley, CA, USA

## Abstract

**Background:**

Tau accumulation in the brain is postulated to follow a hierarchical pattern, known as Braak staging, originally defined in postmortem brains. PET‐based Braak stages have since been proposed, however, tau PET tracers exhibit distinct binding characteristics that might influence the detectable trajectories of tau accumulation and its relationship with other biomarkers across the AD continuum. In a head‐to‐head study, we investigated the emergence of tau positivity in each Braak region, along with plasma *p*‐tau217 and GFAP, as a function of Aβ PET deposition.

**Method:**

We evaluated 352 individuals from the HEAD study (205 cognitively unimpaired and 147 cognitively impaired) with head‐to‐head Aβ PET, MK6240 and Flortaucipir tau PET, and plasma *p*‐tau217 (ALZpath) and GFAP (Quanterix) measures. Tau PET Braak regions, plasma *p*‐tau217 and GFAP trajectories were modeled as functions of Aβ burden (Centiloid scale) using the Lowess method. Biomarkers were z‐scored anchored on young individuals (*n* = 19, <28 years old). Positivity for plasma biomarkers and for each Braak region was determined when values surpassed 2.5 z‐score from the mean.

**Result:**

Among the tested markers, plasma GFAP was the earliest to show abnormality as a function of Centiloid, occurring at 11 Centiloids. For MK6240, Braak I was the first regions to became positive (at 22 Centiloids), followed by Braak II (at 32 Centiloids), Braak IV (at 42 Centiloids), Braak III (at 52 Centiloids), Braak V (at 70 Centiloids) and Braak VI (at 123 Centiloid; Figure 1A). For Flortaucipir the first region to became positive was Braak IV (at 50 Centiloids), followed by Braak I and II (both at 56 Centiloids), Braak III (at 85 Centiloids) and Braak V (at 92 Centiloids). Braak VI region did not reach the threshold for positivity in our population (Figure 1B). Plasma *p*‐tau217 reached the positivity threshold at 38 Centiloids.

**Conclusion:**

MK6240 follows a Braak stage‐like pattern of abnormality as a function of Aβ burden, whereas for Flortaucipir, Braak IV is the first region to became abnormal. MK6240 becomes abnormal at lower Aβ burden levels compared to plasma *p*‐tau217 and Flortaucipir but after plasma GFAP. Our findings suggest that MK6240 might detect tau pathology in earlier disease stages than Flortaucipir.